# The Administrative Side of Sanatorium Work

**Published:** 1909-08-07

**Authors:** 


### Nursing Administration.

### THE ADMINISTRATIVE SIDE OF SANATORIUM WORK.

### II.—THE DOMESTIC STAFF.

It is obvious that in an open air colony containing accommodation for thirty or forty patients there is a great deal of work to be got through. But unless the sanatorium is to be maintained at an enormous cost, the domestic staff must be strictly limited to the number of persons found necessary to supervise the patients in doing everything for themselves. The principle must be established from the outset that the patients are the staff, but in carrying it into practice and producing a sense of ordered tranquillity instead of scramble and discomfort, the co-operation of permanent trained members of the staff must be relied on. The more highly skilled in their special duties these permanent members of the staff become, the easier it will be for the whole household.

The cook is perhaps the most important functionary in the colony next to the matron. She will have to cope with large appetites, and it should be her pride to gratify them at the smallest possible expense. She ought to be an adept in the art of producing variety with plain materials, a good manager, well grounded in the knowledge of food values, and thoroughly practised in the science of economy. It is foolish policy to pare down the cook's salary and thus procure only an uneducated woman, when by securing the right kind of person the sanatorium may easily save as much as two or three hundred a year. A cook trained on good modern lines should receive from £40 to £60 a year, and the position is one to attract an educated woman. She would need a strong permanent kitchenmaid under her, but for much of the routine work in her department she would look to the female patients, and she should be capable of using unskilled labour and turning it to good account. In such an institution, even when life is mainly lived out of doors, there is much scrubbing and swabbing to be done. For this, which is not always suitable work for phthisical patients, it may be advisable to get in outside help, paying by the hour, this being exactly the kind of help which can readily be obtained in any village at small cost. The washing up, which requires special care in a colony of this kind, would be carried out by the patients under the supervision of the cook, who would control all the workers employed in her special department.

The general house-work includes the cleaning and keeping in order of all the living rooms, dormitories, verandahs, and open-air sheds; the serving of meals, attendance on those who are in bed, attendance on the matron and nurses, answering the door, and such general duties as filling lamps, lighting gas, attending to fires, cleaning metal fittings, etc. There must be one head housemaid, with a good head on her shoulders, on whom the matron can depend for such part of the day's routine as cannot well be performed by the patients. It will be her duty to superintend the work of the female patients, both in the dormitories, and in other directions; and it is essential that she should possess a pleasant manner in fulfilling this duty, and be capable of entering into the true spirit of the house. In practice it is sometimes found inadvisable to employ women on the men's side, and there should be a permanent manservant, who may with advantage be an old patient, to undertake the charge of the men's dormitories, supervise the male patients' duties in this department, bring in the coal, etc. It will be seen therefore that the permanent domestic staff need not consist of more than four persons—cook, kitchenmaid, head housemaid, and man. But the success of the whole establishment depends on all being thoroughly efficient. False economy in the direction of wages is responsible for a great degree of unnecessary expense in the end.

When all is told the wages bill of the permanent staff will not be heavy.

| | | |
|---|---|---|
| Cook ... ... | ...£50 | |
| Kitchen-maid ... | ... 18 | |
| Housemaid ... | ... 22 | |
| Man-servant ... | ... 20 | (old patient) |
| Total ... | £110 | |

Reckoning for a household of some fifty persons, this total is small indeed. True it provides only four persons, but then every member of the tiny staff is well chosen, thoroughly to be depended on, and capable of playing a part in the difficult task of utilising a mass of unskilled and continually changing labour in the various departments of the establishment. If the wages were lowered and inferior people were engaged for these posts, it would be impossible, without general waste and confusion, to work the colony with so small a staff. And the additional cost may easily be estimated of adding two or three extra persons, with their board and wages, to the numbers above indicated. An increase in wages is a mere trifle in comparison.

The organisation of the housework is a matter demanding considerable skill and method on the part of the matron. But in every colony containing female patients the difficulty is rather to find work for all those capable of employment than to find workers. A regular routine is speedily established, and if a high standard is maintained the patients take a keen interest in keeping things smart. It is a great mistake to let any impression gain ground that being a working colony nothing matters very much. In the outdoor life slovenliness and slackness should know no place, and all should be kept as trim and taut as on a man-of-war. The strain of employing the patients' labour for domestic duties is most felt in beginning a new colony. As time goes on the matron has always two or three on whom she can depend as knowing the ways of the place, and she may sometimes be able to retain a useful patient at very low wage who is glad to remain on for the good of her health and take entire charge of some branch of the work.

